# Characterization of the complete mitochondrial genome of *Gymnosoma dolycoridis* (Diptera, Tachinidae) and phylogenetic analysis

**DOI:** 10.1080/23802359.2022.2107449

**Published:** 2022-08-04

**Authors:** Rong Wang, Yan Zhi, Qiuyu Yao, Chuntian Zhang, Jiayu Liu

**Affiliations:** a Key Laboratory of Medical Insects, Guizhou Medical University, Guiyang, China; b School of Basic Medical Science, Guizhou Medical University, Guiyang, China; c Laboratory Animal Center, Guizhou Medical University, Guiyang, China; d College of Life Sciences, Shenyang Normal University, Shenyang, China

**Keywords:** *Gymnosoma dolycoridis*, mitochondrial genome, phylogeny, Tachinidae

## Abstract

The complete mitogenome of *Gymnosoma dolycoridis* Dupuis, 1960 was determined in this study. It is 15,185 bp in length, consisting of 13 protein-coding genes (PCG), 22 transfer RNA genes, two ribosomal RNA genes and one non-coding control region. The A + T content of the mitogenome is 78.5%. A maximum-likelihood phylogenetic tree built on 13 PCGs of 15 tachinid species indicated that *Gymnosoma dolycoridis* is clustered with other members of the subfamily Phasiinae as conventional taxonomy predicted.

Tachinidae is one of the largest families of Diptera with 8592 known species (O’Hara et al. 2009, 2020; O’Hara and Henderson 2020). To date, however, the mitogenomes of only nine tachinid species have been reported (Shao et al. 2012; Zhao et al. 2013; Li et al. 2017; Hou et al. 2018, 2019; Pei et al. 2019; Seo et al. 2019; Luo et al. 2021; Yan et al. 2021). Herein, the complete mitogenome of *Gymnosoma dolycoridis* Dupuis, 1960 of Phasiinae was determined and described, which would provide useful genetic information for improving the taxonomic system and phylogenetics of Tachinidae.

The specimen (*Gymnosoma dolycoridis*) was collected from Huaxi District, Guiyang city, Guizhou Province, China (106.620159 N, 26.36755 E) in May 2020 and deposited at the Key Laboratory of Medical Insects of Guizhou Medical University (https://www.gmc.edu.cn/, Jiayu Liu, fsliujiayu@163.com) under the voucher number GD20200520. Total DNA was extracted from thoracic muscle tissues using Rapid Animal Genomic DNA Isolation Kit (Sangon Biotech Co., Ltd., Shanghai, China). Whole genome sequencing was conducted on Illumina HiSeq PE150 platform. Clean data were assembled and annotated by Geneious Prime 2020.2.2 (Kearse et al. 2012) and MITOS Web Server (http://mitos2.bioinf.uni-leipzig.de/index.py) (Bernt et al. 2013). The rRNA and protein-coding genes (PCGs) were identified and confirmed via multiple sequence alignment with homologous genes from published mitochondrial genomes of other species in Tachinidae. The tRNA genes were identified using tRNAscan-SE (Lowe and Eddy 1997).

The assembled mitogenome of *G. dolycoridis* (15,185 bp in length) is available at the NCBI GenBank database under the accession numbers OK631974. The overall nucleotide composition was A (40.2%), T (38.3%), G (8.9%), C (12.6%) and A + T content (78.5%). The complete mitogenome was composed of 13 protein-coding genes (PCGs), two rRNA genes, 22 tRNA genes and one non-coding region. Four PCGs, two rRNA genes and eight tRNA genes were distributed on the light strand among the 38 sequence elements, while others on the heavy strand.

The 13 PCGs accounted for 73.4% of the complete mitogenome of *G. dolycoridis* (11,146 bp). PCGs utilized a variety of start codons including the standard ATN, except for the nonstandard CGA (*COI*). The most frequent start codon was ATG, which was covered six PCGs (*COII*, *ATP6*, *COIII*, *ND4*, *ND4L* and *CYTB*). The stop codon TAA was assigned to most of the PCGs (*ND2*, *ATP8*, *ATP6*, *COIII*, *ND4L* and *ND6*), but an incomplete stop codon T was used by four PCGs (*COI*, *COII*, *ND5* and *ND4*), *ND3*, *CYTB* and *ND1* terminated with the codon TAG.

To investigate the phylogenetic status of *Gymnosoma dolycoridis* in Tachinidae, a maximum-likelihood phylogenetic tree was reconstructed using MEGAX 10.2.6 (Kumar et al. 2018) based on the combined nucleotide sequences of the 13 PCGs for 15 tachinid species. As shown in the phylogenetic tree (Figure 1), Phasiinae species formed a steady monophyletic group with high support, *G. dolycoridis* was clustered in Phasiinae as morphological taxonomy predicted.

**Figure 1.**
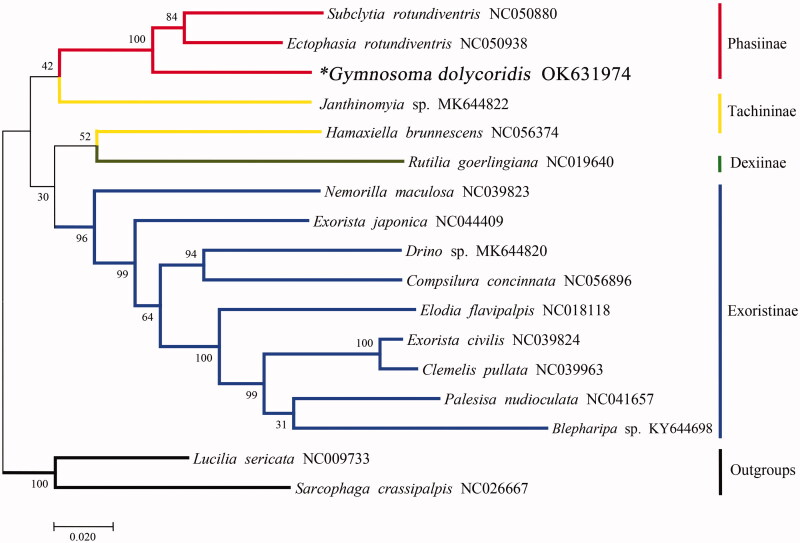
The maximum-likelihood phylogenetic analysis of 15 tachinid species based on the combined 13 protein-coding genes. Accession numbers of mitochondrial sequences used in the phylogenetic analysis are listed after scientific name. *Species documented in this study.

## Data Availability

The genome sequence data that support the findings of this study are openly available in GenBank of NCBI at https://www.ncbi.nlm.nih.gov/ under the accession no. OK631974. The associated BioProject, SRA and Bio-Sample numbers are PRJNA776999, SRR16955181 and SAMN22830838, respectively.
